# Retinal Nerve Fiber Layer Thickness. The Beijing Eye Study 2011

**DOI:** 10.1371/journal.pone.0066763

**Published:** 2013-06-24

**Authors:** Ya Xing Wang, Zhe Pan, Liang Zhao, Qi Sheng You, Liang Xu, Jost B. Jonas

**Affiliations:** 1 Beijing Institute of Ophthalmology, Beijing Tongren Hospital, Capital University of Medical Science, Beijing, China; 2 Department of Ophthalmology, Faculty of Clinical Medicine Mannheim, University of Heidelberg, Mannheim, Germany; Zhongshan Ophthalmic Center, China

## Abstract

**Purpose:**

To measure retinal nerve fiber layer (RNFL) thickness in a population-based setting.

**Methods:**

In the population-based Beijing Eye Study 2011 with 3468 individuals, RNFL thickness was measured in a subgroup of 1654 (47.7%) study participants by spectral domain optical coherence tomography (iVue SD-OCT).

**Results:**

Mean RNFL thickness was significantly (*P*<0.001) higher in the inferior sector (131.4±20.6 µm) than the superior sector (126.1±19.1 µm), where it was higher than in the temporal sector (79.8±12.2 µm;*P*<0.001), where it was higher than in the nasal sector (75.1±12.6 µm;*P*<0.001). In multivariate analysis, mean global RNFL thickness (103.2±12.6 µm) increased significantly with younger age (standardized correlation coefficient beta:−0.30;*P*<0.001), larger neuroretinal rim area (beta:0.26;*P*<0.001), shorter axial length (beta:−0.21*;P*<0.001), thicker subfoveal choroidal thickness (beta:0.15;*P*<0.001), larger optic disc area (beta:0.10;*P*<0.001), less refractive lens power (beta:0.10;*P*<0.001), flatter anterior cornea (beta:0.07;*P* = 0.01) and female gender (beta:0.05;*P* = 0.03). In this population with an age of 50+ years, the age-related decline in RNFL thickness was 0.5 µm per year of life or 0.36% of an original RNFL thickness of 137 µm at baseline of the study at 50 years of age. Mean global RNFL thickness decreased by 2.4 µm for each mm enlargement of axial length.

**Conclusions:**

The RNFL profile shows a double hump configuration with the thickest part in the inferior sector, followed by the superior sector, temporal sector and nasal sector. Factors influencing global RNFL thickness were younger age, larger neuroretinal rim, shorter axial length, thicker subfoveal choroid, larger optic disc, less refractive lens power, flatter anterior cornea and female gender. Beyond an age of 50+ years, RNFL decreased by about 0.3% per year of life at an age of 50+ years and by 2.4 µm per mm of axial elongation. These findings may be of interest for the knowledge of the normal anatomy of the eye and may be of help to diagnose diseases affecting the RNFL.

## Introduction

The retinal nerve fiber layer (RNFL) is located between the inner retinal limiting membrane as basal lamina of the Müller cells and the retinal ganglion cell layer. The RNFL consists of the retinal ganglion cell axons, which are covered by astrocytes and bundled by processes of Müller glial cells. The examination of the RNFL is of high importance for the diagnosis of optic nerve anomalies and diseases, since the retinal ganglion cell axons continue into the optic nerve fibers behind the optic nerve head. Examination of the RNFL may even be helpful to get information about the brain, since the retinal ganglion cells and their axons have direct contact with the brain and can be regarded as an outpost of the brain outside of the interior cavity of the cranium. The RNFL can be assessed by conventional ophthalmoscopy [1], on wide-angle fundus photographs [2], by confocal scanner laser tomography [3], scanning laser polarimetry [4], and optical coherence tomography (OCT) [5], [6]. With the clinical introduction of the new generation of spectral domain OCTs, the OCT based examination of the RNFL has become one of the most important tools to assess the status of the optic nerve. Since normative data of the RNFL thickness measured by the new generation of OCTs in population-based studies have been scarcely available so far [7], we conducted this study to measure the RNFL thickness by spectral domain OCT in the population-based Beijing Eye Study. A population-based study in contrast to a hospital-based investigation has the advantage to be free of a potential selection bias by the referring ophthalmologists.

## Methods

### Ethics

The Medical Ethics Committee of the Beijing Tongren Hospital approved the study protocol and all participants gave informed written consent, according to the Declaration of Helsinki.

The Beijing Eye Study 2011 with a population-based cross-sectional design was performed in urban communities in the Haidian district in the North of Central Beijing and in rural communities in the village area of Yufa of the Daxing District south of Beijing [8], [9]. Inclusion criterion was an age of 50+ years. Out of 4403 eligible individuals, 3468 individuals (1963 (56.6%) women) participated in the eye examination, corresponding to an overall response rate of 78.8%. The study was divided into a rural part (1633 (47.1%) subjects; 943 (57.7%) women) and an urban part (1835 (52.9%) subjects; 1020 (55.6%) women). The mean age was 64.6±9.8 years (median, 64 years; range, 50–93 years). Trained research technicians asked the study participants questions providing information on demographic variables, socioeconomic background, and known major systemic diseases. Cognitive function was assessed using the MMSE (mini mental state examination) scale [10]. Psychic depression was assessed using the Zung Self-rating Depression Scale assessment test [11]. Fasting blood samples were taken for measurement of blood lipids, glucose and glycosylated hemoglobin HbA1c. Blood pressure was measured. Body height and weight and the circumference of the waist and hip were recorded. The ophthalmic examination included measurement of presenting visual acuity and uncorrected visual acuity. Best corrected visual acuity was assessed by automatic refractometry (Auto Refractometer AR-610, Nidek Co., Ltd, Tokyo, Japan), if uncorrected visual acuity was lower than 1.0. Intraocular pressure was measured by pneumotonometry. A slit lamp examination carried out by an experienced ophthalmologist assessed lid abnormalities, Meibomian gland dysfunction, corneal disorders, and peripheral anterior chamber depth using van Herick’s method. Using optical low-coherence reflectometry (Lensstar 900® Optical Biometer, Haag-Streit, 3098 Koeniz, Switzerland), biometry of the right eyes was performed for measurement of the anterior corneal curvature, central corneal thickness, anterior chamber depth, lens thickness and axial length. The pupil was dilated using tropicamide once or twice, until the pupil diameter was at least 6 mm. Digital photographs of the cornea and lens and retro-illuminated photographs of the lens were taken using the Neitz CT-R camera (Neitz Instruments Co., Tokyo, Japan). Monoscopic photographs of the macula and optic disc were taken using a fundus camera (Type CR6-45NM, Canon Inc. U.S.A.). The optic nerve head, peripapillary area, and macula were scanned by two spectral-domain OCTs (iVue SD-OCT; Optovue Inc. Fremont, CA, U.S.A.; Spectralis, Heidelberg Engineering, Heidelberg, Germany).

RNFL thickness was measured by spectral domain OCT (iTVue SD-OCT; Optovue Inc. Fremont, CA, U.S.A.) in a randomized subgroup of the study population. The iVue scan mode was similar to RTVue-100, as described previously in detail [12], [13]. The manufacturer’s Glaucoma Protocol, 3-D Optic Disc Protocol, iWellness Protocol was applied. This Glaucoma Protocol measured the circumpapillary RNFL thickness by recalculating data along a circle of 3.45 mm in diameter around the optic disc, created by a scan pattern made up of 13 concentric circles with diameters from 1.3 to 4.9 mm with 0.3 mm intervals and 12 radial lines with diameters of 3.40 mm centered on the optic nerve head. The sampling rate was 26,000 A-scans/second, resulting in 14,241 A-scans acquired in 0.55 seconds. The software’s automatic detection of the optic nerve head margins was reviewed and manually adjusted to ensure proper centering over the optic disc prior to analysis. Mean global RNFL thicknesses provided by the iVue system were recorded and re-checked by calculating the mean of the RNFL thickness in all four sectors.

Only those subjects with OCT measurements of the RNFL thickness were included into the study. Statistical analysis was performed using a commercially available statistical software package (SPSS for Windows, version 20.0, IBM-SPSS, Chicago, IL). In a first step, we determined the mean value (presented as mean ± standard deviation) of the main outcome parameter. In a second step, we performed univariate analyses of the associations between RNFL thickness and other ocular and systemic parameters. In a the third step, we carried out multivariate regression analyses with RNFL thickness as the dependent parameter and all systemic parameters as independent variables which were associated significantly with RNFL thickness in the univariate analyses. In a fourth step, the multivariate analysis additionally included all ocular parameters, which were associated significantly with RNFL thickness in the univariate analyses. We performed an analysis of collinearity and calculated the variance inflation factors (VIF). All *P*-values were two-sided.

## Results

Measurements of the RNFL thickness were available for 1654 (47.7%) study participants (938 (56.7%) women). The group of subjects without RNFL thickness measurements as compared with the group of subjects with RNFL thickness measurements was significantly older (63.1±9.5 years versus 66.2±9.9 years; *P*<0.001) and had significantly shorter axial length (23.1±1.1 mm versus 23.4±1.1 mm; *P*<0.001). Both groups did not vary significantly in gender (women/men: 1024/790 or 56.5% versus 938/716 or 56.7% *P* = 0.89) and refractive error (−0.70±2.06 diopters versus −0.81±2.19 diopters; *P* = 0.12). The mean age in our study population with RNFL thickness measurements was 66.2±9.9 years (median: 66 years; range: 50–93 years), the mean refractive error was −0.81±2.19 diopters (median: −0.38 diopters; range: −21.0 to +6.00 diopters), and the mean axial length was 23.4±1.1 mm (median: 23.3 mm; range: 20.29 mm –30.36 mm). With the scan quality criteria of SSI ≥30, accepted measurements were obtained in 1625 eyes (98.2% of all scans).

**Figure 1:**
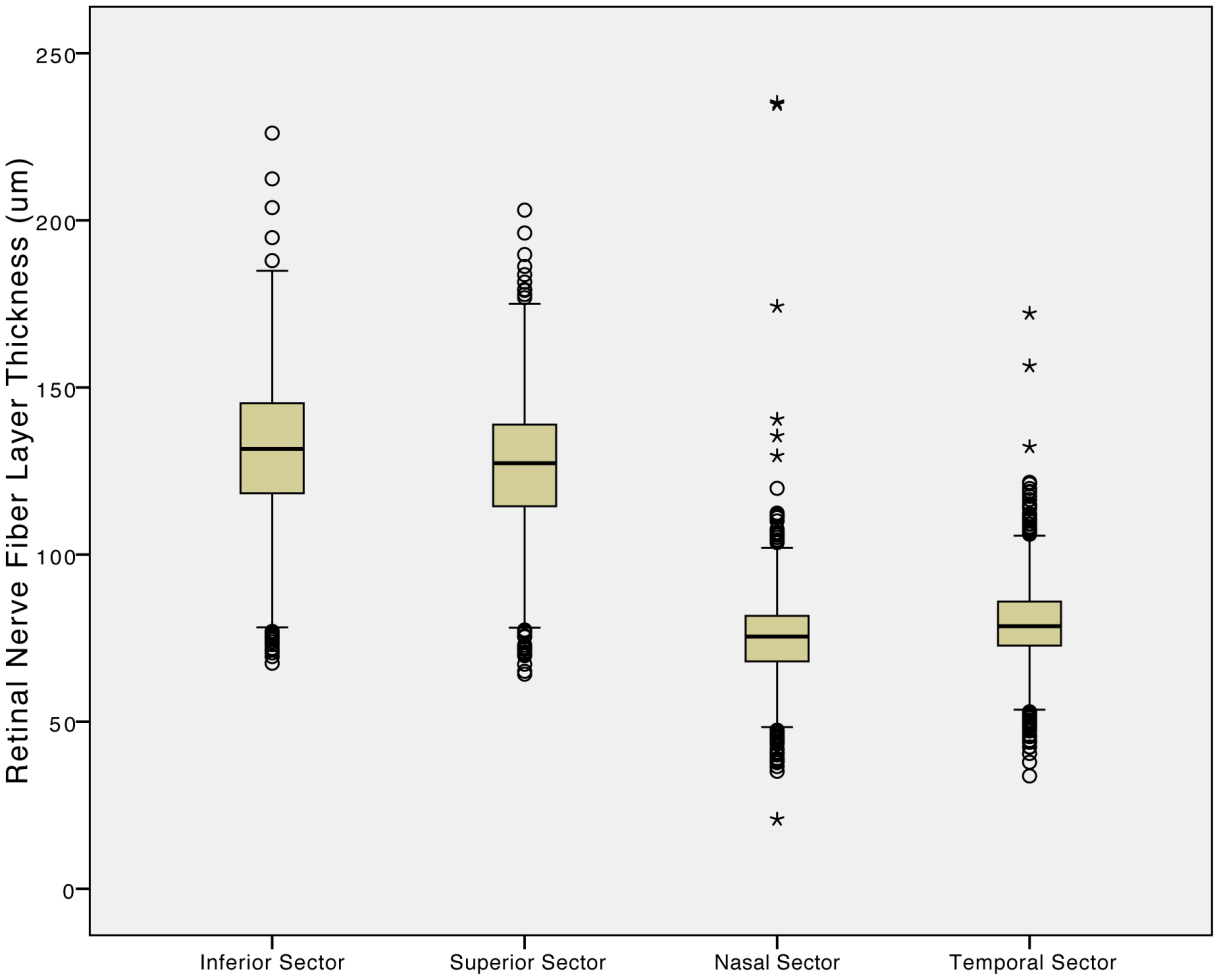
Boxplots showing the distribution of retinal nerve fiber layer thickness measured by spectral domain optical coherence tomography (iVue SD-OCT) in four parapapillary sectors in the Beijing Eye Study 2011.

**Figure 2:**
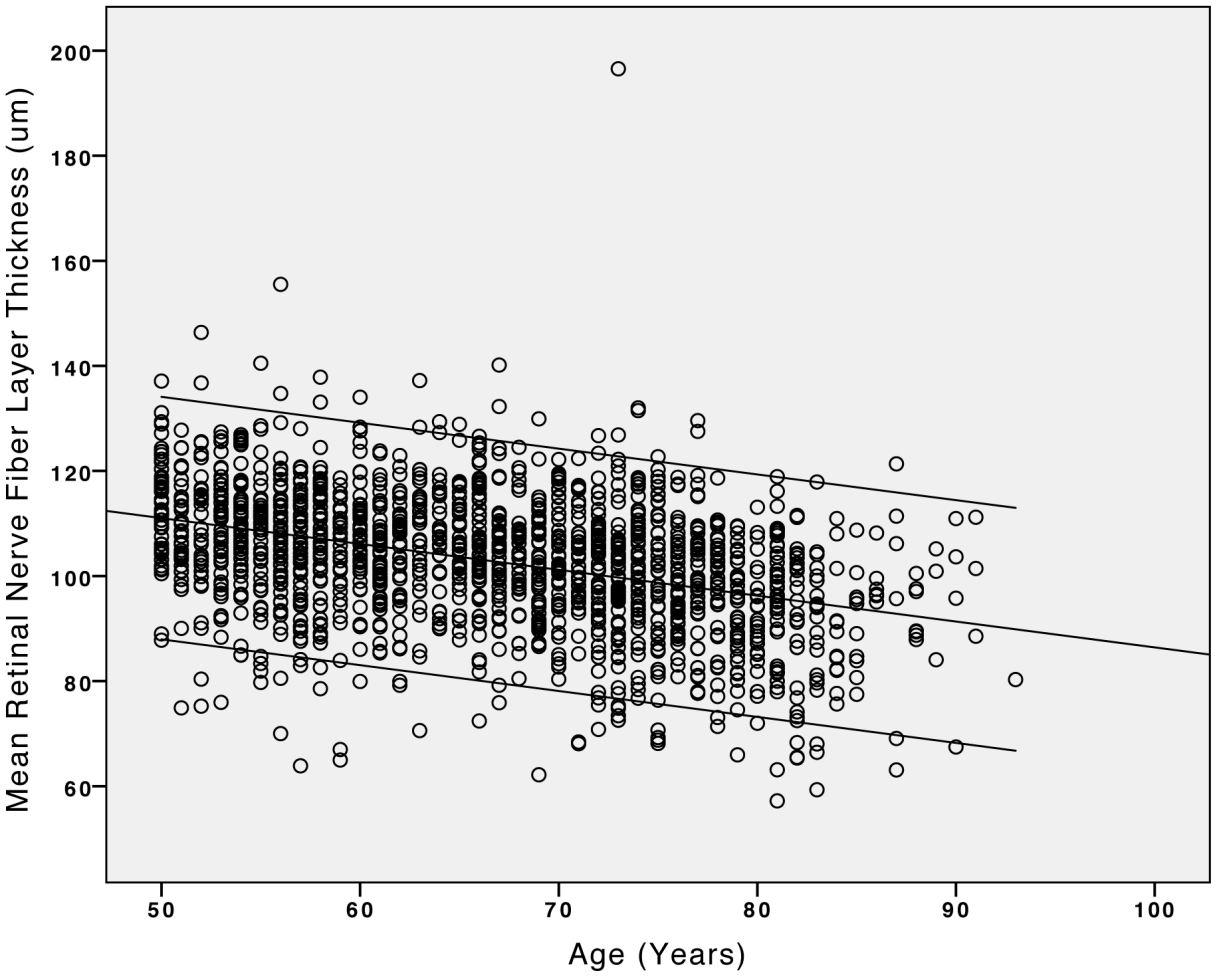
Scattergram showing the correlation (and 95% confidence interval of the regression line) between age and the mean retinal nerve fiber layer thickness measured by spectral domain optical coherence tomography (iVue SD-OCT) in the Beijing Eye Study 2011.

**Figure 3:**
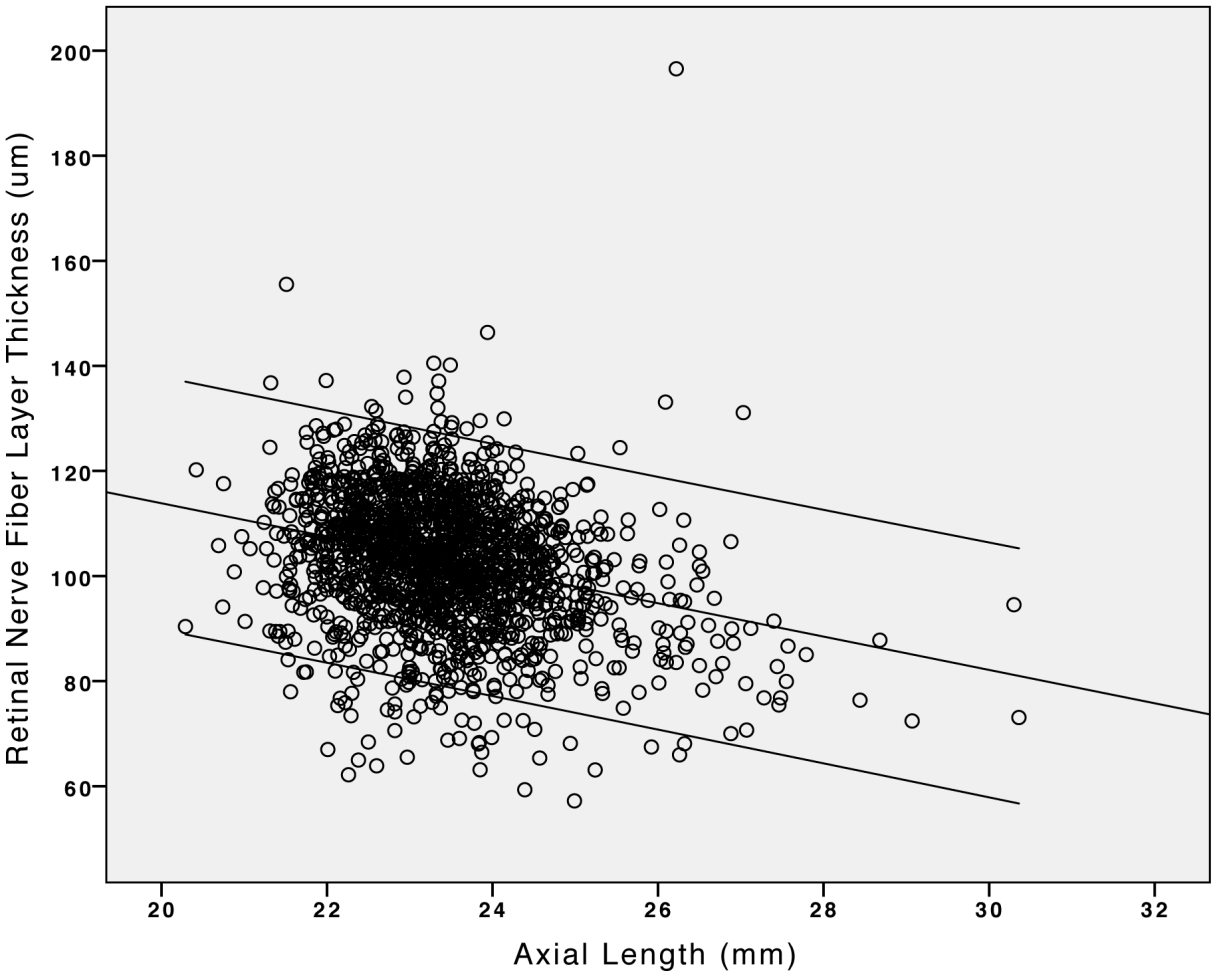
Scattergram showing the correlation (and 95% confidence interval of the regression line) between axial length and the mean retinal nerve fiber layer thickness measured by spectral domain optical coherence tomography (iVue SD-OCT) in the Beijing Eye Study 2011.

Mean RNFL thickness was significantly (*P*<0.001) higher in the inferior sector (131.4±20.6 µm) than in the superior sector (126.1±19.1 µm) where it was significantly (*P*<0.001) higher than in the temporal sector (79.8±12.2 µm), where it was significantly (*P*<0.001) higher than in the nasal sector (75.1±12.6 µm) (Fig. 1). Mean global RNFL thickness was 103.2±12.6 µm (median: 103.9 µm; range: 57.2 µm - 196.5 µm). In univariate analysis, mean global RNFL thickness decreased significantly with systemic parameters such as older age (Fig. 2), higher body mass index, higher cognitive score, and higher prevalence of diabetes mellitus and arterial hypertension, and with ocular parameters such as higher best corrected visual acuity, hyperopic refractive error, shorter axial length (Fig. 3), larger area of the optic disc and neuroretinal rim, smaller alpha zone and smaller beta zone of parapapillary atrophy, and thicker subfoveal choroid, to mention only a few (Tables 1,2). It was not significantly associated with rural versus urban region of habitation, body height and weight, depression score, smoking, alcohol consumption, snoring, mean blood pressure, blood concentrations of glucose, HbA1c and high-density lipoproteins, central corneal thickness, intraocular pressure, ocular perfusion pressure, scleral spur distance and corneal diameter (Tables 1,2).

**Table 1:** Associations between mean global retinal nerve fiber layer thickness as measured by spectral domain optical coherence tomography (iVue SD-OCT) and ocular and systemic parameters in the Beijing Eye Study 2011 (univariate analysis).

Parameter	*P*-Value	Regression Coefficient B (or Mean Difference)	Standardized Coefficient Beta	95% Conf. Interval of B or Mean Difference)
Age (Years)	<0.001	−0.49	−0.38	−0.55, −0.43
Gender	<0.001	3.24	0.13	2.00, 4.47
Rural/Urban Region	<0.001	−4.25	−0.14	−5.70, −2.80
Body Height (cm)	0.230			
Body Weight (kg)	0.05	0.05	0.05	0, 0.10
Body Mass Index (kg/m^2^)	0.001	0.28	0.08	0.11, 0.45
Waist Circumference (cm)	0.12			
Cognitive Score	<0.001	0.38	0.09	0.17, 0.58
Depression Score	0.95			
Level of Education	0.04	−0.70	−0.05	−1.37, 0.03
Smoking	0.32			
Package Years	0.54			
Alcohol Consumption	0.39			
Aspirin Intake	0.006	−1.84	−0.07	−3.16, 0.05
Snoring	0.859			
Systolic Blood Pressure (mmHg)	0.002	−0.05	−0.08	−0.09, −0.02
Diastolic Blood Pressure (mmHg)	0.001	0.10	0.08	0.04, 0.15
Mean Blood Pressure (mmHg)	0.66			
Blood Concentrations of:Glucose (mmol/L)	0.50			
HbA1c (%)	0.09			
High-Density Lipoproteins (mmol/L)	0.90			
Low-Density Lipoproteins (mmol/L)	0.001	1.39	0.10	0.56, 2.22

**Table 2:** Associations between mean global retinal nerve fiber layer thickness as measured by spectral domain optical coherence tomography (iVue SD-OCT) and ocular and systemic parameters in the Beijing Eye Study 2011 (univariate analysis).

Parameter	*P*-Value	Regression Coefficient B (or Mean Difference)	Standardized Coefficient Beta	95% Conf. Interval of B or Mean Difference
Cholesterol (mmol/L)	0.007	1.03	0.08	0.28, 1,78
Prevalence of: Diabetes Mellitus	<0.001	3.95		3.19, 5.70
Arterial Hypertension	<0.001	3.12		1.86, 4.36
Best Corr. Visual Acuity (logMAR)	<0.001	−25.2	−0.26	−29.7, −20.7
Refractive Error (Diopters)	<0.001	1.40	0.23	1.11, 1.68
Axial Length (mm)	<0.001	−3.18	−0.28	−3.71, −2.64
Ant. Corneal Curvature Radius (mm)	0.008	−4.36	−0.09	−6.71, −2.00
Central Corneal Thickness (µm)	0.06			
Anterior Chamber Depth (mm)	<0.001	−2.42	−0.10	−3.58, −1.27
Lens Thickness (mm)	<0.001	−3,94	−0.11	−5.81, −2.06
Lens Refractive Power (Diopters)	<0.001	1.07	0.18	0.76, 1.38
Optic Disc Area (mm^2^)	<0.001	5.25	0.17	3.75, 6.74
Neuroretinal Rim Area (mm^2^)	<0.001	10.74	0.34	9.32, 12.16
Horizontal Cup/Disc Diameter Ratio	<0.001	−6.30	−0.13	−8.63, −3.97
Vertical Cup/Disc Diameter Ratio	<0.001	−13.0	−0.24	−15.56, −10.51
Parapapillary Atrophy (mm^2^)				
Alpha Zone	0.01	−1.50	−0.07	−2.64, −0.35
Beta Zone	<0.001	−1.96	−0.18	−2.57, −1.35
Choroidal Thickness (µm)	<0.001	0.04	0.35	0.04, 0.05
Intraocular Pressure (mmHg)	0.20			
Ocular Perfusion Pressure (mmHg)	0.80			
Corneal Diameter	0.27			
Scleral Spur Distance (mm)	0.52			
Pupil Diameter (mm)	0.06			
Pupil Distance (mm)	0.11			

The multivariate analysis included RNFL thickness as dependent variable and all systemic parameters as independent variables which were significantly (*P*<0.10) associated with RNFL thickness in the univariate analysis. It revealed that RNFL thickness remained to be significantly associated with lower age (*P*<0.001) and female gender (*P* = 0.003), while body mass index (*P* = 0.16), cognitive function score (*P* = 0.27), aspirin intake (*P* = 0.64), systolic blood pressure (*P* = 0.84), diastolic blood pressure (*P* = 0.78), and blood concentrations of low-density lipoproteins (*P* = 0.20) and cholesterol (*P* = 0.35), and prevalence of diabetes mellitus (*P* = 0.39) and arterial hypertension (*P* = 0.34) were no longer associated with RNFL thickness. In the second part of the multivariate analysis, we included all ocular parameters as independent variables which were significantly associated with RNFL thickness in the univariate analyses, after adjusting for age and gender. We then removed step by step all ocular parameters, which were no longer significantly associated with RNFL thickness, starting with the parameters with the highest *P*-value. It showed that alpha zone (*P* = 0.86) and beta zone (*P* = 0.75) of parapapillary atrophy and pupil diameter (*P* = 0.61), and then refractive error (*P* = 0.09) were no longer significantly associated with RNFL thickness. We then performed an analysis of collinearity and removed the parameters vertical cup/disc ratio (variance inflation factor (VIF): 5.0), horizontal cup/disc ratio (VIF: 4.50), anterior chamber depth (VIF: 2.29) and lens thickness (VIF = 2.27). In the final analysis, RNFL thickness remained to be significantly correlated with lower age (*P*<0.001), female gender, (*P* = 0.03), shorter axial length (*P*<0.001), flatter anterior corneal curvature (*P* = 0.01), less refractive lens power (*P*<0.001), larger optic disc area (*P*<0.001), larger neuroretinal rim area (*P*<0.001), and thicker choroidal thickness (*P*<0.001) (Table 3). The parameters with the highest standardized correlation coefficients beta (and thus the most marked influence on RNFL thickness) were younger age (beta: −0.30), larger neuroretinal rim area (beta: 0.26) and shorter axial length (beta: −0.21), followed by thicker subfoveal choroidal thickness (beta: 0.15), larger optic disc area (beta: 0.10), and less refractive less power (beta = 0.10) (Table 3).

**Table 3:** Associations between mean global retinal nerve fiber layer thickness as measured by spectral domain optical coherence tomography (iVue SD-OCT) and ocular and systemic parameters in the Beijing Eye Study 2011 (multivariate analysis).

	P-Value	Regression Coefficient B	Standardized Coefficient Beta	95%CI of B		Variance Inflation Factor
Age (Years)	<0.001	−0.39	−0.30	−0.45	−0.32	1.34
Gender (Men/Women)	0.03	1.33	0.05	0.17	2.48	1.15
Axial Length (mm)	<0.001	−2.40	−0.21	−3.18	−1.62	2.65
Anterior Corn. Curvature Radius (mm)	0.01	3.33	0.07	0.67	5.98	1.59
Refractive Lens Power (Diopters)	<0.001	−0.58	−0.10	−0.91	−0.26	1.55
Optic Disc Area (mm^2^)	<0.001	3.28	0.10	1.74	4.71	1.18
Neuroretinal Rim Area (mm^2^)	<0.001	8.03	0.26	6.64	9.42	1.11
Subfoveal Choroidal Thickness (µm)	<0.001	0.02	0.15	0.011	0.023	1.53

Using the regression coefficients of the multivariate analysis (Table 3) and assuming a linear age-related decline in the RNFL thickness, global RNFL thickness decreased by about 0.39 µm per life of year at an age of 50+ years (or about 0.35% out of the mean global RNFL thickness of 113 µm at the age of 50 years. If instead the equation of the regression line of the univariate analysis was taken (RNFL thickness (µm) =  −0.49× Age (Years) +136.6 µm), one would expect a loss of 0.49 µm (or 0.36%) for year of life beyond an age of 50+ years. In a similar manner, mean global RNFL thickness decreased by approximately 2.4 µm for each mm enlargement of axial length (Table 1, 2, 3).

## Discussion

Using spectral domain OCT technology in our population-based study on subjects with an age of 50+ years, mean global RNFL thickness (103.2±12.6 µm) was significantly associated with younger age, larger neuroretinal rim area, shorter axial length, thicker subfoveal choroidal thickness, larger optic disc area, less refractive less power, flatter anterior corneal curvature and female gender. In the same sequence, the influence of the parameters on RNFL thickness decreased. In this population with an age of 50+ years, the age-related decline in RNFL thickness was 0.5 µm per year of life or 0.36% of an original RNFL thickness of 137 µm at baseline of the study at 50 years of age. RNFL thickness was unevenly distributed with the highest values in the inferior sector followed by the superior sector, the temporal sector and finally the nasal sector.

The value of the mean global RNFL thickness of 103.2±12.6 µm as found in our study agrees with previous studies on other ethnic groups. In the multi-ethnic study Girkin and colleagues, the mean global RNFL thickness as measured by the spectral domain OCT ranged between 101.9±9.8 µm for the European descent study population and 112.4±9.8 µm for the Hispanic group, with the values for the African descent group, the Indian group and the Japanese groups within that range [14]. In the population-based Singapore Chinese Eye Study using another SD-OCT instrument (Cirrus HD-OCT; Carl Zeiss Meditec, Inc, Dublin, CA) on 542 subjects with a mean age of 53.0±6.4 years (range: 40–80 years), mean global RNFL thickness was 123.0±15.9 µm [7].

The uneven distribution of the RNFL thickness with the highest values in the inferior sector, followed by the superior sector, the temporal sector and finally the nasal sector has already been described in a similar manner in previous studies. In a histomorphometric study by Varma and colleagues on 10 eyes of white individuals with a mean age of 53.1±19.6 years, mean superior, inferior, nasal, and temporal RNFL thickness at the disc margin was 405 µm, 376 µm, 372 µm, and 316 µm, respectively [15]. In a histomorphometric study by Dichtl and coworkers on 22 normal human eyes, the RNFL at the optic disc border showed a double hump configuration with the highest mean thickness in the inferior quadrant (266±64 µm), followed by the superior quadrant (240±57 µm), the nasal quadrant (220±70 µm), and finally the temporal quadrant (170±58 µm) [16]. Interestingly in the latter study, in eyes with absolute glaucoma, mean thickness of the remaining RNFL was 40±18 µm with no marked differences between the disc regions. The uneven distribution of the RNFL thickness corresponds with the distribution of the visibility of the retinal nerve fiber layer on red-free fundus photographs. The RNFL visibility was significantly higher in the temporal inferior fundus region, followed by the temporal superior fundus region, the nasal region and finally the temporal region [17]. It corresponded with the distribution of the diameter of the retinal arterioles, which were significantly in the temporal inferior fundus region, followed by the temporal superior fundus region, the nasal region and finally the temporal region [18]. It also corresponded with the physiologic neuroretinal rim shape following the so-called ISNT (Inferior-Superior-Nasal-Temporal-)rule [19]. The explanations for these spatial correlations may be the location of the fovea about 0.5 mm inferior to the horizontal optic disc axis [17]. Also in studies using the spectral-domain OCT technology, as the Singapore Chinese Eye Study, reported a thicker RNFL in the inferior sector (126.8±16.2 µm) than in the superior sector (123.0±15.9 µm), the temporal sector (71.6±11.2 µm) and finally the nasal sector (69.2±10.8 µm) [7].

The age-related decline in the RNFL thickness has also been reported in previous studies. In the aforementioned Singapore Chinese Eye Study, the average RNFL thickness decreased with older age (*P* = 0.001) after adjustment for optic disc area (*P*<0.001), and axial length (*P*<0.001) [7]. In a similar manner, Sung and coworkers found a the mean loss in global RNFL thickness of 0.26 µm per year of life [20]. It agrees with histomorphometric studies which showed an age-related loss in the number of optic nerve fibers of about 0.3% per year of life at an age of 50+ years [21], [22]. The age-related loss in RNFL thickness also corresponds to the age-dependent loss in the visibility of RNFL as observed clinically and on fundus photographs [17]. The rate of 0.3% loss of RNFL thickness per year of life also fits well with a yearly loss of about 0.3% of retinal rods and cones and of retinal pigment epithelium cells [23], [24].

The association between the RNFL thickness and optic disc size confirms the recent Singapore Chinese Eye Study [7], in which the average RNFL thickness increased with optic disc area. It is again in agreement with a histomorphometric study in which the number of retrobulbar optic nerve fibers increased with the size of the optic disc [25]. As an analogy, the number of retinal photoreceptors increased with optic disc size in non-highly myopic eyes [26]. The association between RNFL thickness and shorter axial length as found in our study was also reported in the Singapore Chinese Eye Study [7]. In contrast to our study, optic disc area had the strongest effect on measurements of RNFL thickness in the Singapore Chinese Eye Study, while in our study younger age, larger neuroretinal rim and shorter axial length had a more marked influence on the RNFL thickness (Table 3). As expected, RNFL thickness was significantly associated with neuroretinal rim area, after adjustment for optic disc size. The neuroretinal rim is the intrapapillary equivalent of the retinal nerve fiber layer and gets lost in glaucomatous optic neuropathy, parallel to a decrease in the RNFL. The associations between RNFL thickness and younger age, shorter axial length and larger optic disc size were also found in hospital-based studies using other spectral domain OCTs [27], [28]. The results of the current study also agree with the findings obtained in a parallel investigation on a similar study population which was examined by another spectral domain OCT device (Spectralis HRA+OCT, Heidelberg Engineering, Heidelberg, Germany) (own data).

Our study is limited by several factors. First, the Beijing Eye Study 2011 had a reasonable response rate of 78.8%, however, differences between participants and non-participants could have led to a selection artefact. Also, the RNFL was measured for a subset of the total study population only, so that, although unlikely, a bias may have occurred. Second, RNFL thickness was examined only in the right eye of each study participant, so that inter-eye differences and their associations with inter-eye differences of other parameters could not be assessed. Third, as any population-based study, our investigation included all eligible and participating subjects from the study region. It indicates that also patients with diseases, such as disorders of the optic nerve and macula were included, although these diseases may have affected the RNFL thickness. Future studies may address whether these diseases were associated with abnormalities of choroidal thickness. Fourth, the eligibility criterion for inclusion into our study was an age of 50+ years. All findings and conclusions made in the study are therefore valid only for the population with an age of 50 or more years. This may be particularly important if one calculates a correlation between age and the RNFL thickness. The linear relationship described for the association between age and RNFL thickness is thus valid only for the population aged 50+ years. To cite an example, one may easily assume that up to an age of about 50 years, the RNFL thickness may be almost constant, and that the age-related loss may start at the age of 50 years then showing a linear relationship. The equation of the linear regression cannot simply be extrapolated to age groups younger than 50 years.

In conclusion, the RNFL profile in a population-based study showed a double hump configuration with the thickest value in the inferior sector, followed by the superior sector, the temporal sector and the nasal sector. Main factors influencing mean global RNFL thickness were younger age, larger neuroretinal rim, shorter axial length, thicker subfoveal choroid, larger optic disc, less refractive lens power, flatter anterior cornea and female gender. Beyond an age of 50+ years, RNFL decreased by about 0.3% per year of life at an age of 50+ years and by 2.4 µm per mm of axial elongation. These findings may be of interest for the knowledge of the normal anatomy of the eye and may be of help to diagnose diseases affecting the RNFL.
